# Bioactivity of brassica seed meals and its compounds as ecofriendly larvicides against mosquitoes

**DOI:** 10.1038/s41598-023-30563-6

**Published:** 2023-03-09

**Authors:** Lina B. Flor-Weiler, Robert W. Behle, Mark A. Berhow, Susan P. McCormick, Steven F. Vaughn, Ephantus J. Muturi, William T. Hay

**Affiliations:** 1 U.S. Department of Agriculture, Agricultural Research Service, National Center for Agricultural Utilization Research, Crop BioProtection Research Unit, 1815 N University St., Peoria, IL 61604 USA; 2 U.S. Department of Agriculture, Agricultural Research Service, National Center for Agricultural Utilization Research, Functional Foods Research Unit, 1815 N University St., Peoria, IL 61604 USA; 3 U.S. Department of Agriculture, Agricultural Research Service, National Center for Agricultural Utilization Research, Mycotoxin Prevention and Applied Microbiology Research Unit, 1815 N University St., Peoria, IL 61604 USA

**Keywords:** Natural products, Entomology

## Abstract

Strategic, sustainable, and ecofriendly alternatives to chemical pesticides are needed to effectively control mosquitoes and reduce the incidence of their vectored diseases. We evaluated several *Brassicaceae* (mustard family) seed meals as sources of plant derived isothiocyanates produced from the enzymatic hydrolysis of biologically inactive glucosinolates for the control of *Aedes* *aegypti* (L., 1762). Five defatted seed meals (*Brassica* *juncea* (L) Czern., 1859, *Lepidium* *sativum* L., 1753, *Sinapis* *alba* L., 1753, *Thlaspi* *arvense* L., 1753, and *Thlaspi* *arvense*—heat inactivated and three major chemical products of enzymatic degradation (allyl isothiocyanate, benzyl isothiocyanate and 4-hydroxybenzyl isothiocyanate) were assayed to determine toxicity (LC_50_) to *Ae.* *aegypti* larvae. All seed meals except the heat inactivated *T.* *arvense* were toxic to mosquito larvae. *L.* *sativum* seed meal was the most toxic treatment to larvae (LC_50_ = 0.04 g/120 mL dH_2_O) at the 24-h exposure. At the 72-h evaluation, the LC_50_ values for *B.* *juncea,* *S.* *alba* and *T.* *arvense* seed meals were 0.05, 0.08 and 0.1 g/120 mL dH_2_O, respectively. Synthetic benzyl isothiocyanate was more toxic to larvae 24-h post treatment (LC_50_ = 5.29 ppm) compared with allyl isothiocyanate (LC_50_ = 19.35 ppm) and 4-hydroxybenzyl isothiocyanate (LC_50_ = 55.41 ppm). These results were consistent with the higher performance of the benzyl isothiocyanate producing *L.* *sativum* seed meal. Isothiocyanates produced from seed meals were more effective than the pure chemical compounds, based on calculated LC_50_ rates. Using seed meal may provide an effective method of delivery for mosquito control. This is the first report evaluating the efficacy of five *Brassicaceae* seed meals and their major chemical constituent against mosquito larvae and demonstrates how natural compounds from *Brassicaceae* seed meals can serve as a promising ecofriendly larvicides to control mosquitoes.

## Introduction

Vector-borne diseases caused by aedine mosquitoes remain a critical global public health challenge. Incidence of mosquito-borne diseases are spreading geographically^1–3^ and have reemerged causing severe disease outbreaks^4–7^. The spread of diseases in humans and animals (i.e., chikungunya, dengue fever, Rift Valley Fever, yellow fever and Zika) is unprecedented. For dengue alone, approximately 3.6 billion people in the tropics are at risk of infection with estimated 390 million annual infections causing a range of 6100–24,300 deaths annually^8^. The reemergence of Zika virus causing outbreak in South America captivated global attention by causing brain damage to children born of infected women^2^. Kraemer et al.^3^ predicted that *Aedes* mosquitoes will further expand geographically and by 2050, half of the world’s population will be at risk of mosquito transmitted arboviruses.

Vaccines have yet to be developed for most mosquito-borne diseases, except those recently developed for dengue and yellow fever^9–11^. Availability of these vaccines remains limited and are administered for clinical trials only. Targeting mosquito vectors using synthetic insecticides has been the key control strategy to prevent mosquito-borne disease transmission^12,13^. Although effective for killing mosquitoes, continued use of synthetic insecticides has had a negative impact to non-target organisms and has polluted the environment^14–16^. More concerning is the trend of increasing insecticide resistance to chemical insecticides by mosquitoes^17–19^. These problems associated with insecticides have accelerated the search for effective and ecofriendly alternative vector controls.

A variety of plants have been exploited as sources of botanical insecticides for control of insect pests^20,21^. Botanicals are typically ecologically safe because they are biodegradable and have low or negligible toxicity to non- target organisms such as mammals, fishes, and amphibians^20,22^. Botanicals are known to produce diverse bioactive compounds with varied modes of action to effectively control different life stages of mosquitoes^23–26^. The plant-derived compounds such as essential oils and other active botanical components have gained attention and opened the way for innovative tools for managing mosquito vectors. Essential oils, monoterpenes and sesquiterpenes function as repellents, feeding deterrents and ovicides^27–33^. Many plant oils cause death of larval, pupal, and adult stages of mosquitoes^34–36^, targeting their nervous, respiratory, endocrine and other critical systems of insects^37^.

Recent studies have provided insights on the potential use of plants from the mustard family Brassicaceae, and their seed meals as sources of bioactive compounds. Mustard seed meals have been tested as biofumigants^38–41^ and applied as soil amendment to suppress weed growth^42–44^, and control soilborne plant pathogens^45–50^, plant feeding nematodes^41,51–54^ and insect pests^55–60^. The biocidal activity of these seed meals is attributed to the class of plant defense compounds known as isothiocyanates^38,42,60^. *In* *planta* these defense compounds are stored within the plant cell as non-bioactive glucosinolates. However, when the plant is damaged by insect feeding or pathogen infection, the glucosinolates are hydrolyzed by myrosinase enzymes to bioactive isothiocyanates^55,61^. Isothiocyanates are volatile compounds known to have a broad-spectrum antimicrobial and insecticidal activity that vary substantially in structure, biological activity, and content among Brassicaceae species^42,59,62,63^.

Although isothiocyanates produced from mustard seed meals are known to be insecticidal, data on bioactivity against medically important arthropod vectors is lacking. Our study examined the larvicidal activity of four defatted seed meals against *Ae.* *aegypti* larvae. The aim of the study was to evaluate their potential use as ecofriendly biopesticides for mosquito control. Three major chemical constituents from seed meals, allyl isothiocyanate (AITC), benzyl isothiocyanate (BITC) and 4-hydroxybenzyl isothiocyanate (4-HBITC) were also tested to verify the bioactivity of these chemical components against mosquito larvae. This is the first report evaluating the efficacy of four brassica seed meals and their major chemical constituent against mosquito larvae.

## Materials and methods

### Source of mosquitoes

A laboratory colony of *Aedes* *aegypti* (Rockefeller strain) was maintained at 26 °C, 70% relative humidity (RH) and 10:14 h (L:D photoperiod). Mated females held in plastic cages (11 cm high × 9.5 cm diameter) were fed via an artificial feeding system using citrated bovine blood (HemoStat Laboratories Inc., Dixon, CA, USA). Blood feeding was done routinely using membrane style multiple glass feeders (Chemglass, Life Sciences LLC, Vineland, NJ, USA) attached via tubing to a circulating water bath (HAAKE S7, Thermo-Scientific, Waltham, MA, USA) set at 37 °C. A parafilm M membrane was stretched over the base of the inner chamber of each glass feeder (154 mm^2^ area). Each feeder was then positioned on the top mesh covering a cage containing mated females. Approximately 350–400 μL of bovine blood was added to the funnel of the glass feeder using a Pasteur pipet (Fisherbrand, Fisher Scientific, Waltham, MA, USA) and the adults were allowed to blood feed for at least an hour. Gravid females were then provided with 10% sucrose solution and allowed to oviposit eggs on moist filter paper lining the inside of a solo ultra clear souffle cup (1.25 Fl. Oz size, Dart Container Corp., Mason, MI, USA) half filled with water placed inside the cage. Filter papers with eggs were placed in Ziploc bags (SC Johnsons, Racine, WI) and stored at 26 °C. Eggs were hatched and batches of approximately 200–250 larvae were reared in plastic trays and larvae were fed with a mixture of rabbit food (ZuPreem, Premium Natural Products, Inc., Mission, KS, USA), liver powder (MP Biomedicals, LLC, Solon, OH, USA) and fish flakes (TetraMin, Tetra GMPH, Mell, Germany) at 2:1:1 ratio. Late third instar larvae were used for our bioassays.

### Source of plant seed materials

Plant seed materials used in this study were obtained from the following commercial and government sources: *Brassica* *juncea* (Brown Mustard-Pacific Gold) and *Sinapis* *alba* (White Mustard—Ida Gold) from Pacific Northwest Farmers Cooperative, Spokane WA, USA; *Lepidium* *sativum* (Garden Cress) from Kelly Seed and Hardware Co., Peoria, IL, USA; and *Thlaspi* *arvense* (Field Pennycress—Elisabeth) from USDA-ARS, Peoria, IL, USA. All seeds used in the study were not treated with pesticides for seed treatment. Processing and utilization of all seed materials in this study followed the local and national regulations in accordance with all relevant local State and national guidelines. There were no genetically modified plant cultivars examined in this study.

### Seed meal production

Seeds from *B.* *juncea* (PG), *L.* *sativum* (*Ls*), *S.* *alba* (IG), *Thlaspi* *arvense* (DFP), were ground to a fine meal using a Retsch ZM200 ultra centrifugal mill (Retsch, Haan, Germany), equipped with a 0.75 mm screen and a stainless steel 12 tooth rotor at 10,000 rpm (Table 1). The ground seed meal was transferred to a paper thimble and defatted using hexane in a Soxhlet apparatus for 24-h. A subsample of the defatted field pennycress was heat treated at 100 °C for 1-h to denature the myrosinase enzymes and prevents hydrolysis of the glucosinolate to form the bioactive isothiocyanates. The heat-treated *T.* *arvense* seed meal (DFP-HT) was used as a negative control treatment by denaturing the myrosinase enzyme.

**Table 1:** Glucosinolate concentration in ground defatted seed meals after Soxhlet extraction.

Species	Common name	Cultivar	Predominant glucosinolate
*Brassica* *juncea* (PG)	Brown mustard	Pacific gold	Sinigrin (33.3 ± 1.5 mg/g)
*Thlaspi* *arvense* (DFP)	Pennycress	Elisabeth	Sinigrin (26.5 ± 0.9 mg/g)
*Lepidium* *sativum* (*Ls*)	Garden cress	–	Glucotropaeolin (36.6 ± 1.2 mg/g)
*Sinapis* *alba* (IG)	White mustard	Ida gold	Sinalbin (38.0 ± 0.5 mg/g)

### Liquid chromatography

The glucosinolate content of defatted seed meals were determined in triplicate using high-performance liquid chromatography (HPLC) following previously reported protocols^64^. Briefly, 3 mL of methanol was added to a 250 mg sample of defatted seed meal. Each sample was sonicated in a water bath for 30 min and let stand in the dark at 23 °C for 16 h. A 1 mL aliquot of the organic layer was then filtered through a 0.45 µm filter into an auto sampler vial. Seed meal glucosinolate content was determined in triplicate on a Shimadzu HPLC System (two LC 20AD pumps; SIL 20A autoinjector; DGU 20As degasser; SPD-20A UV–VIS detector monitoring at 237 nm; and a CBM-20A communication BUS module) running under the Shimadzu LC solutions Version 1.25 software (Shimadzu Corporation, Columbia, MD, USA). The column was a C_18_ Inertsil reverse phase column (250 mm X 4.6 mm; RP C-18, ODS-3, 5u; GL Sciences, Torrance, CA, USA). The initial mobile phase conditions were set to 12% methanol/88% aqueous 0.01 M tetrabutylammonium hydroxide (TBAH; Sigma-Aldrich, St. Louis, MO, USA) at a flow rate of 1 mL/min. After the injection of a 15 µl of sample, the initial conditions were held for 20 min, and then the solvent ratios were adjusted up to 100% methanol for a total sample run time of 65 min. Freshly prepared standards of sinalbin, glucotropaeolin, and sinigrin (Sigma-Aldrich, St. Louis, MO, USA) were serially diluted to make the standard curves (nM/mAbs basis) used to evaluate the concentration of glucosinolates in defatted seed meals. Sample glucosinolate concentrations were validated on an Agilent 1100 HPLC (Agilent, Santa Clara, CA, USA) running OpenLAB CDS ChemStation edition (C.01.07 SR2[255]) equipped with the same column and performing the previously stated methodology; glucosinolate concentrations were found to be comparable between HPLC systems.

### Synthetic isothiocyanates

Allyl isothiocyanate (94%, stab.) and benzyl isothiocyanate (98%) were purchased from Fisher Scientific (Thermo Fisher Scientific, Waltham, MA, USA). The 4-hydroxybenzyl isothiocyanate was purchased from ChemCruz (Santa Cruz Biotechnology, CA, USA). The glucosinolates sinigrin, glucotropaeolin and sinalbin produce allyl isothiocyanate, benzyl isothiocyanate and 4-hydroxybenzyl isothiocyanate, respectively, when enzymatically hydrolyzed by myrosinase enzymes.

### Larvicidal bioassays

Laboratory bioassays followed the methods by Muturi et al.^32^ with modifications. Five defatted seed meal treatments DFP, DFP-HT, IG, PG and Ls were used in the study. Twenty larvae were introduced into 400 mL disposable tri-pour beakers (VWR International, LLC, Radnor, PA, USA) with 120 mL deionized water (dH_2_O). Toxicity of seed meals to mosquito larvae were tested at seven concentrations: 0.01, 0.02, 0.04, 0.06, 0.08, 0.1 and 0.12 g of seed meal/120 mL dH_2_O for DFP, DFP-HT, IG and PG seed meals. Preliminary bioassays indicated that defatted *Ls* seed meal was more toxic compared with the other four seed meals tested. Thus, we adjusted the seven treatment concentrations for *Ls* seed meal with the following concentrations: 0.015, 0.025, 0.035, 0.045, 0.055, 0.065 and 0.075 g/120 mL dH_2_O.

A no treatment control group (dH_2_0 without seed meal additives) was included to assess normal insect mortality under assay conditions. Toxicological bioassays for each seed meal consisted of three replicate tri-pour beakers (20 late third instar larvae per beaker) for a total of 108 containers. The treated containers were held at room temperature (20–21 °C) and larval mortality was recorded at 24- and 72-h of continuous exposure to treatment concentrations. A mosquito larva was considered dead if the body and appendage did not move when prodded or touched with a fine stainless-steel spatula. Dead larvae generally remain motionless at the bottom of the container or surface of the water in either dorsal or ventral position. The experiment was repeated three times on different days using different cohorts of larvae for a total of 180 larvae exposed to each treatment concentration.

AITC, BITC and 4-HBITC were evaluated for toxicity to mosquito larvae using the same bioassay procedure but with a different treatment application. A stock solution for each chemical component at 100,000 ppm was prepared by adding 100 µL of chemical to 900 µL absolute ethanol in a 2-mL centrifuge tube and vortexed for 30 s to mix thoroughly. Treatment concentrations were determined based on our preliminary bioassays where BITC was found to be far more toxic than AITC and 4-HBITC. Toxicity assays used five concentrations of BITC (1, 3, 6, 9, and 12 ppm), seven concentrations for AITC (5, 10, 15, 20, 25, 30 and 35 ppm) and 6 concentrations for 4-HBITC (15, 30, 45, 60, 75 and 90 ppm). The control treatment received 108µL of absolute ethanol, which is equivalent to the largest volume with the chemical treatment. The bioassay was replicated and repeated as described above exposing a total of 180 larvae for each concentration of a treatment. Larval mortality for each AITC, BITC and 4-HBITC concentration was recorded after 24-h of continuous exposure to treatments.

### Statistical analysis

Dosage-response mortality data were subjected to Probit analysis^65^ using Polo software (Polo Plus, LeOra Software, version 1.0) to calculate the 50% lethal concentration (LC_50_), 90% lethal concentration (LC_90_), slope, lethal dose ratios and 95% confidence intervals for the lethal dose ratios based on log-transformed concentrations and dose-mortality curves. Mortality data were based on combined replication data to provide 180 larvae exposed to each treatment concentration. Probit analysis was conducted separately for each seed meal and each chemical constituent. Toxicity of seed meals and chemical constituents to mosquito larvae were considered significantly different based on the 95% confidence intervals of the lethal dose ratios such that the confidence interval that included a value of 1 were not significantly different, *P* = 0.05^66^.

## Results

HPLC results determining the predominant glucosinolate from the defatted seed meals DFP, IG, PG and *Ls* is presented in Table 1. The predominant glucosinolates differed among the seed meals tested, except for DFP and PG, which both contained sinigrin glucosinolate. The sinigrin content was greater in PG than DFP at 33.3 ± 1.5 and 26.5 ± 0.9 mg/g, respectively. The *Ls* seed meal contained glucotropaeolin at 36.6 ± 1.2 mg/g while IG seed meal had sinalbin at 38.0 ± 0.5 mg/g.

**Figure 1:**
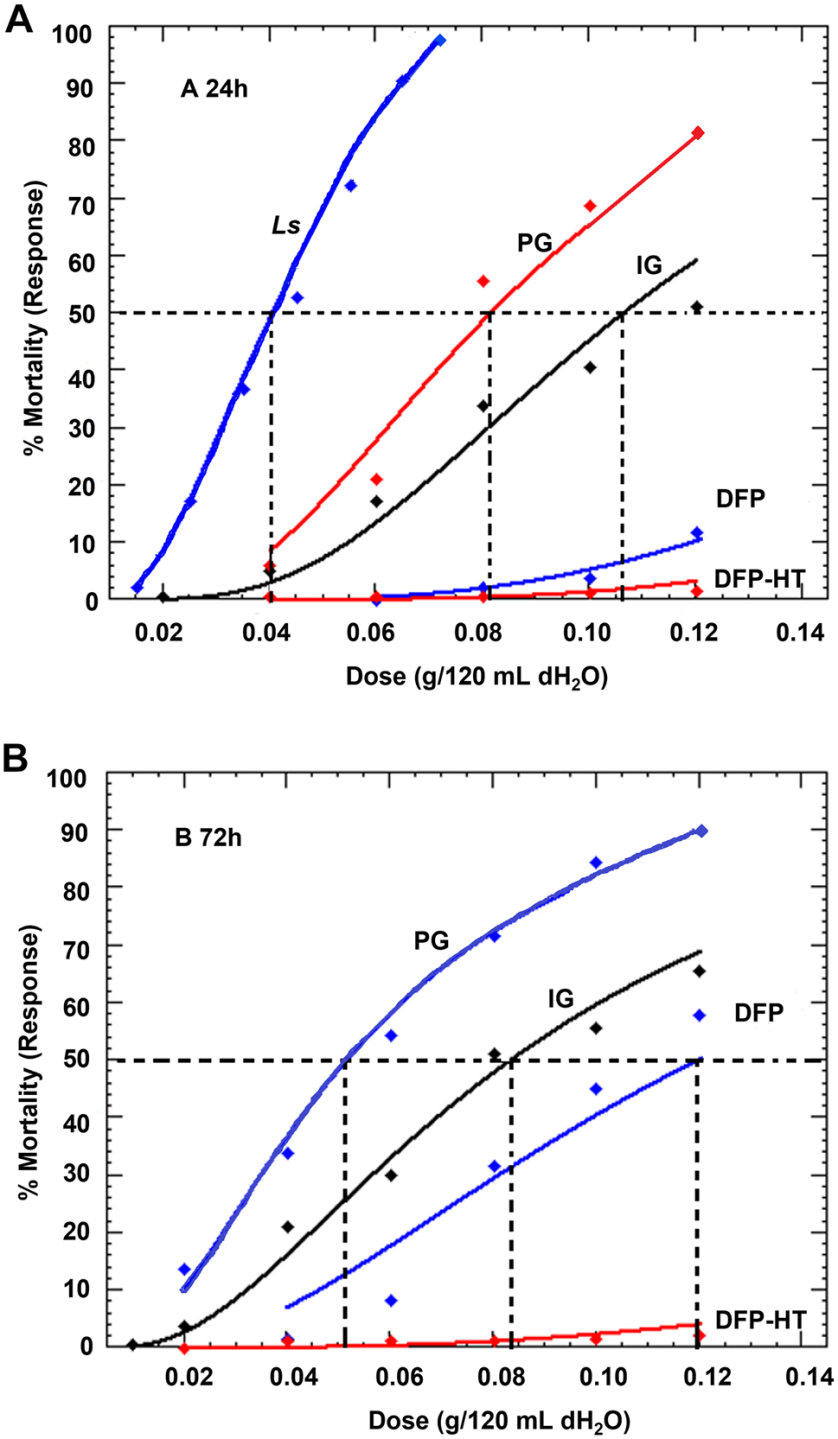
Mortality curve estimated by the dose–response (Probit) of *Ae.* *aegypti* larvae (3rd larval instars) to seed meal concentrations 24 h (**A**) and 72 h (**B**) post treatment. Broken lines represent the LC_50_ of seed meal treatments. DFP *Thlaspi* *arvense*, DFP-HT *Thlaspi* *arvense* heat inactivated, IG *Sinapsis* *alba* (Ida Gold), PG *Brassica* *juncea* (Pacific Gold), *Ls* *Lepidium* *sativum*.

Larvae of *Ae.* *aegypti* were killed when exposed to defatted seed meal treatments, although treatment efficacy varied by plant species. Only the DFP-HT was not toxic to mosquito larvae after 24- and 72-h exposures (Table 2). Toxicity of active seed meals increased with increasing concentrations (Fig. 1A,B). Toxicity of seed meals to mosquito larvae were significantly different based on the 95% CI of the lethal dose ratios of LC_50_ values at 24- and 72-h evaluations (Table 3). At 24-h, the toxic effect of *Ls* seed meal was greater than the other seed meal treatments, providing the fastest activity and greatest toxicity to larvae (LC_50_ = 0.04 g/120 mL dH_2_O). Larvae had low susceptibility to DFP at 24-h with statistically higher LC_50_ value of 0.211 g/120 mL dH_2_O as compared with IG, *Ls* and PG seed meal treatments with LC_50_ values of 0.115, 0.04 and 0.08 g/120 mL dH_2_O, respectively (Table 3). The LC_90_ values were 0.376, 0.275, 0.137 and 0.074 g/120 mL dH_2_O for DFP, IG, PG and *Ls*, respectively (Table 2). The highest concentration of DFP, at 0.12 g/120 mL dH_2_O, caused a mean larval mortality of only 12% after 24-h evaluation while IG and PG was as high as 51 and 82% mean larval mortality, respectively. The highest concentration treatment for *Ls* seed meal (0.075 g/120 mL dH_2_O) had a 99% mean larval mortality after 24-h evaluation (Fig. 1A).

**Table 2:** Toxicity of Brassicaceae seed meals (DFP—*Thlaspi* *arvense*; DFP-HT—*Thlaspi* *arvense* heat inactivated; IG—*Sinapis* *alba* (Ida Gold); PG—*Brassica* *juncea* (Pacific Gold; *Ls—Lepidium* *sativum*) reported as LC_50_ and LC_90_ values for mortalities of late 3rd instar *Aedes* *aegypti* evaluated after 24 and 72 h of continuous treatment exposure.

H	Seed meal	g/120 mL dH_2_O		Slope ± SE	χ^2^	df
		LC_50_ (95% CI)	LC_90_ (95% CI)			
24	DFP	0.211 (0.182–0.277)	0.376 (0.284–0.635)	5.119 ± 0.805	1.7	4
	DFP-HT^a^	–	–	–	–	–
	IG	0.115 (0.106–0.126)	0.275 (0.230–0.353)	3.372 ± 0.280	2.1	4
	PG	0.080 (0.069–0.096)	0.137 (0.108–0.245)	5.451 ± 0.419	3.9	3
	Ls^b^	0.040 (0.037–0.044)	0.074 (0.064–0.091)	4.935 ± 0.281	9.4	4
72	DFP	0.111 (0.101–0.125)	0.215 (0.177–0.298)	4.475 ± 0.306	8.6	4
	DFP-HT^a^	–	–	–	–	–
	IG	0.085 (0.079–0.092)	0.254 (0.215–0.312)	2.706 ± 0.178	3.6	3
	PG	0.051(0.044–0.059)	0.138 (0.108–0.206)	2.978 ± 0.205	4.9	4

^a^Not effective as larval treatment. Data did not generate LC_50_ and LC_90_ values.

^b^*Ls—Lepidium* *sativum—*all dead at higher concentrations after 24 h continuous exposure.

**Table 3:** LC_50_ lethal dose ratio values with corresponding 95% CI showing significant differences between seed meals 24 and 72 h of continuous treatment exposure to *Ae.* *aegypti* larvae.

H	Seed meal	LC_50_ Lethal dose ratios (95% CI)		
		DFP	IG	PG
24	IG	1.89 (1.503–2.363)		
	PG	2.66 (1.175–3.249)	1.44 (1.310–1.592)	
	*Ls*	5.06 (4.136–6.196)	2.75 (2.487–3.041)	0.51 (0.477–0.541)
72	IG	1.30 (1.171–1.451)		
	PG	2.10 (1.903–2.320)	1.61 (1.437–1.810)	

Lethal dose ratio of a seed meal treatment in a column compared with a seed meal treatment in a row are statistically different from each other when the 95% CI of the LC_50_ lethal dose ratio values did not include a value of 1.0, (*P* = 0.05).

At the 72-h evaluation, the LC_50_ values for DFP, IG and PG seed meals were 0.111, 0.085 and 0.051 g/120 mL dH_2_O, respectively. Larvae exposed to *Ls* seed meal were nearly all dead after 72 h exposure such that mortality data did not fit the Probit analysis. Larvae were less susceptible to DFP seed meal treatments with statistically higher LC_50_ value compared with the other seed meals (Tables 2, 3). The LC_50_ values after 72-h evaluations were 0.111, 0.085 and 0.05 g/120 mL dH_2_O for DFP, IG and PG seed meal treatments, respectively. The LC_90_ values after 72-h evaluation were 0.215, 0.254 and 0.138 g/120 mL dH_2_O for DFP, IG and PG seed meals, respectively. Mean larval mortality for DFP, IG and PG seed meal treatments at the highest concentration of 0.12 g/120 mL dH_2_O after 72-h evaluation were 58, 66 and 96%, respectively (Fig. 1B). After 72-h evaluation, the PG seed meal was more toxic compared with both IG and DFP seed meals.

**Figure 2:**
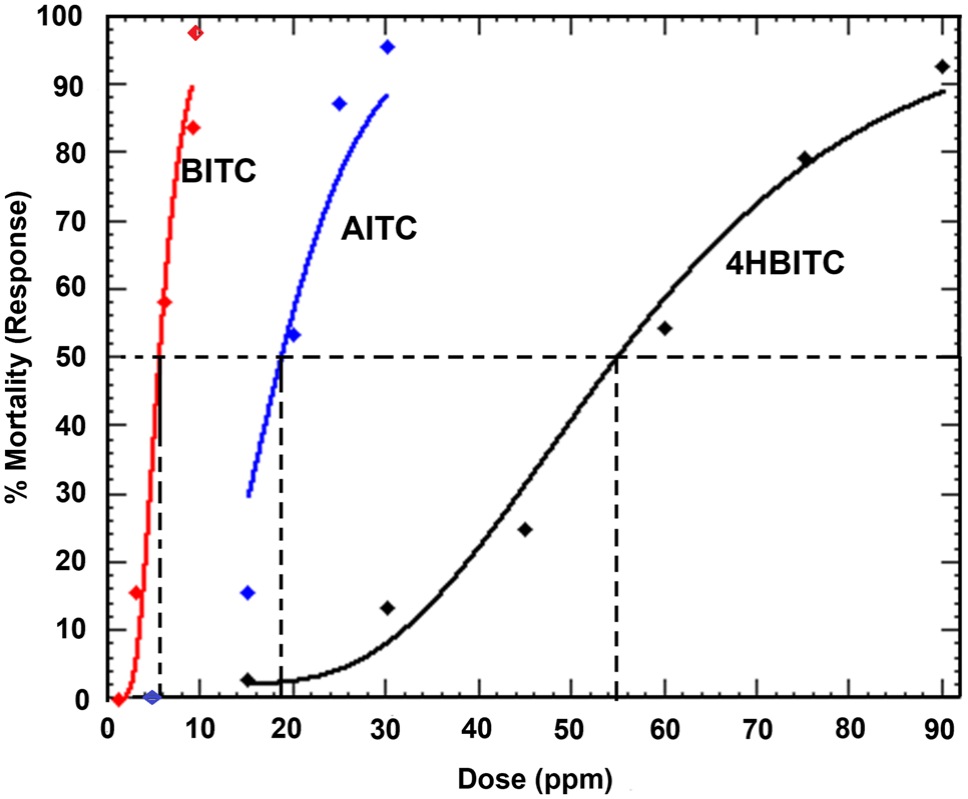
Mortality curve estimated by the dose response (Probit) of *Ae.* *aegypti* larvae (3rd larval instars) to synthetic isothiocyanate concentrations 24 h post treatment. Broken lines represent the LC_50_ of isothiocyanate treatments. *BITC* benzyl isothiocyanate, *AITC* allyl isothiocyanate and 4-HBITC.

Synthetic isothiocyanates, allyl isothiocyanate (AITC), benzyl isothiocyanate (BITC) and 4-hydroxybenzyl isothiocyanate (4-HBITC), effectively killed mosquito larvae. The BITC was more toxic to larvae 24-h post treatment with an LC_50_ value of 5.29 ppm compared with AITC at 19.35 ppm and 4-HBITC at 55.41 ppm (Table 4). The 4-HBITC was less toxic compared to AITC and BITC with higher LC_50_ value. Toxicity to mosquito larvae varied significantly between the two predominant isothiocyanates of the most effective seed meals, *Ls* and PG. Toxicities based on the lethal dose ratios for LC_50_ values between AITC, BITC and 4-HBITC indicated statistical differences where the 95% CI of the LC_50_ lethal dose ratios did not include a value of 1 (*P* = 0.05, Table 4). When evaluated, the highest concentrations of both BITC and AITC killed 100% of test larvae (Fig. 2).

**Table 4:** LC_50_ and LC_90_ values of major chemical component of Brassica seed meal 24 h post treatment to late 3rd instar larvae of *Aedes* *aegypti*.

Compound	Ppm		Slope ± SE	χ^2^	df
	LC_50_ (95% CI)	LC_90_ (95% CI)			
Allyl isothiocyanate (AITC)	19.35 (18.77–19.90) a	26.53 (25.55–27.79)	9.34 ± 0.58	1.21	2
Benzyl isothiocyanate (BITC)	5.29 (4.95–5.64) b	10.61 (9.57–12.10)	4.23 ± 0.31	0.33	2
4-Hydroxy benzyl isothiocyanate (4HBITC)	55.41c (46.65–62.54) c	91.79 (78.6–126.51)	5.85 ± 0.43	12.0	3

LC_50_ values for treatments followed by the same letter are not significantly different based on the lethal dose ratio value in which the 95% confidence interval (CI) does not include a value of 1 (*P* = 0.05).

LC_50_ lethal dose ratio between AITC and BITC = 3.66 (3.404–3.935).

LC_50_ lethal dose ratio between AITC and 4HBITC = 0.34 (0.325–0.365).

LC_50_ lethal dose ratio between BITC and 4HBITC = 0.094 (0.087–0.102).

## Discussion

The use of plant-based bioinsecticides for mosquito control has long been explored as a vector management tool^67,68^. Many plants produce natural chemicals that offer insecticidal activity^37^. Their bioactive compounds offer an attractive alternative to synthetic insecticides with great potential to control insect pests including mosquitoes^26,28,30–35^.

Mustard plants are grown as crops to produce seeds that are used as a spice and a source of oil. When mustard oil is extracted from seeds, or in the case of pennycress extracted for use as a biofuel^69^, one byproduct is the defatted seed meal. This seed meal retains many of its natural biochemistries and hydrolyzing enzymes. Toxicities of these seed meals are attributed to the production of isothiocyanates^55,60,61^. Isothiocyanates are produced from hydrolysis of glucosinolates by the enzyme myrosinase when the seed meal is hydrated^38,55,70^ and are known to have fungicidal, bactericidal, nematocidal, and insecticidal effects as well as other attributes including allelopathic and chemotherapeutic properties^61,62,70^. Several studies reported that mustard plants and seed meals effectively acted as fumigants to suppress soil and stored product insect pests^57,59,71,72^. In this study, we evaluated four seed meals and three of their bioactive products, AITC, BITC and 4-HBITC for toxicity against larvae of *Ae.* *aegypti*. Adding the seed meals directly to the water with the mosquito larvae was expected to activate the enzymatic process to produce the isothiocyanates that would be toxic to mosquito larvae. This biological conversion was partly validated by the observed larvicidal activity of the seed meals and the loss of insecticidal activity when the pennycress seed meal was heat treated prior to application. Heat treatment is expected to destroy the hydrolyzing enzyme to activate glucosinolates preventing the formation of bioactive isothiocyanates. This is the first study to document insecticidal properties of brassica seed meals against mosquitoes in an aqueous environment.

Among the seed meals tested, garden cress (*Ls*) seed meal was most toxic, providing significant mortality of *Ae.* *aegypti* larvae in 24-h continuous treatment. The other three seed meals (PG, IG and DFP) had slower activity, still causing significant mortality after 72-h of continuous treatment. Only *Ls* seed meal contained substantial amount of glucotropaeolin while PG and DFP had sinigrin and IG had sinalbin as the predominant glucosinolates (Table 1). Glucotropaeolin is hydrolyzed into BITC and sinalbin is hydrolyzed into 4-HBITC^61,62^. The results of our bioassays demonstrated consistent high toxicity of both the *Ls* seed meal and the synthetic BITC against mosquito larvae. PG and DFP seed meals contain sinigrin as the predominant glucosinolate content, which hydrolyses to AITC. AITC effectively killed mosquito larvae with an LC_50_ value of 19.35 ppm. The isothiocyanate 4-HBITC is the least toxic to larvae compared to AITC and BITC. Although AITC was less toxic than BITC, both of their LC_50_ values were lower than those of many essential oils tested against mosquito larvae^32,73–75^.

Brassica seed meals we used against mosquito larvae contained one predominant glucosinolate, accounting for more than 98–99% of total glucosinolates, as determined via HPLC. Trace amounts of other glucosinolates were detected but amounted to less than 0.3% of the total glucosinolates. Garden cress (*L.* *sativum*) seed meal had a secondary glucosinolate (sinigrin) but was 1% of the total glucosinolates, still of negligible amount (approximately 0.4 mg/g seed meal). While PG and DFP had the same predominant glucosinolate (sinigrin), the larvicidal activity of their seed meals differed significantly based on their LC_50_ values. Different toxicities to *Ae.* *aegypti* larvae may have been due to differences in myrosinase activity or stability between these two seed meals. The activity of myrosinase enzyme plays an important role in the bioavailability of hydrolysis products like isothiocyanates among Brassicaceae plants^76^. Previous reports of Pocock et al.^77^ and Wilkinson et al.^78^ have demonstrated that variabilities of myrosinase activity and stability can also be attributed to genetic and environmental factors.

**Figure 3:**
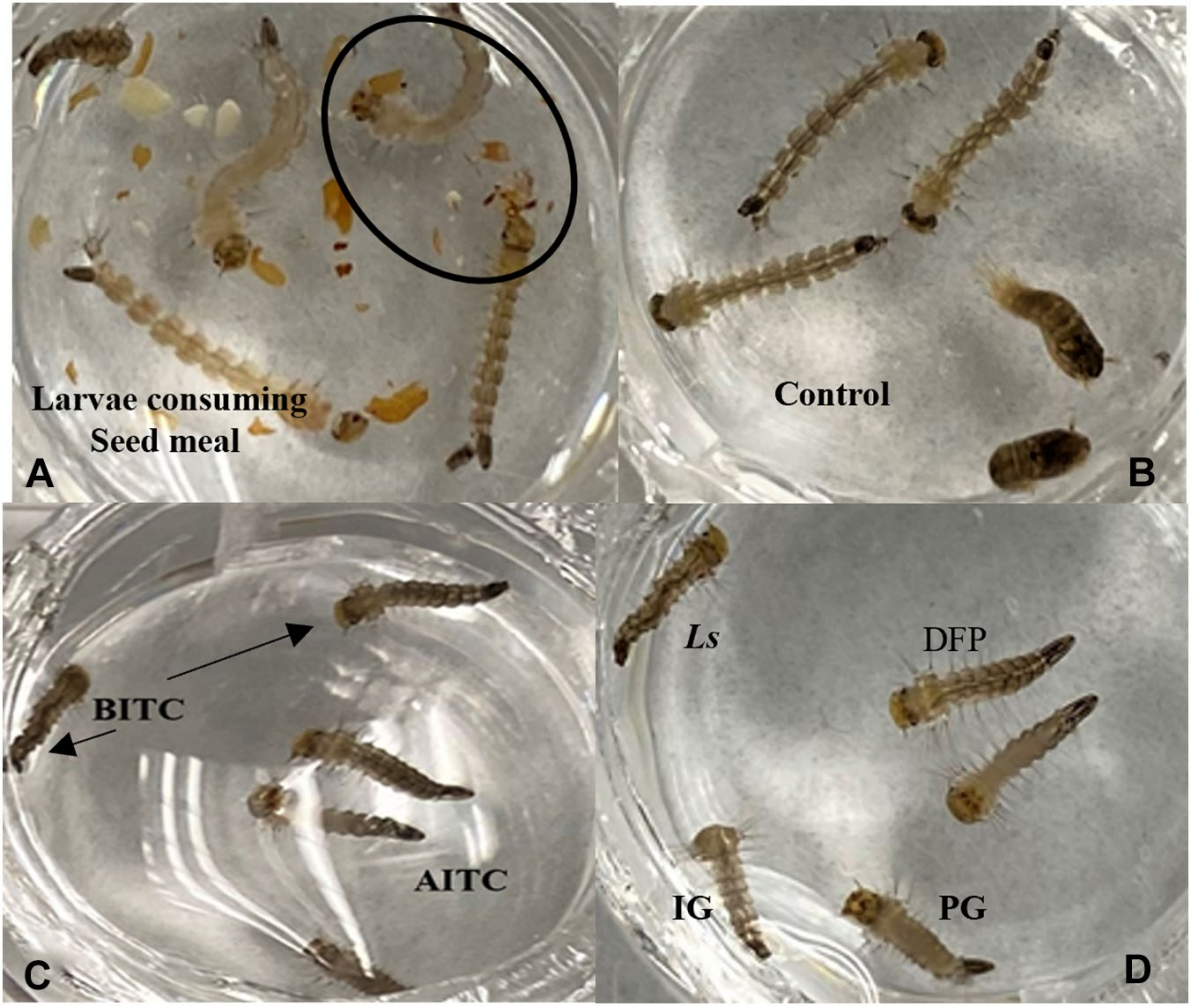
Larvae of *Ae.* *aegypti* exposed continuously to brassica seed meals for 24- to 72-h. (**A**) Dead larvae with seed meal particles in their mouthparts (encircled); (**B**) control treatment (dH_2_0 without seed meal additives) showing normal larval growth and started pupating after 72-h; (**C,D**) seed meal treated larvae exhibiting developmental differences and failed to pupate.

We calculated the expected amount of the bioactive isothiocyanate from the LC_50_ values for each seed meal at 24- and 72-h (Table 5) for comparison with respective chemical applications. Isothiocyanates from seed meals at 24 h appeared to be more toxic than the pure chemical compounds. The LC_50_ values based on estimated isothiocyanates in parts per million (ppm) for seed meal treatments are all below the LC_50_ values of BITC, AITC and 4-HBITC applications. We observed that the larvae consumed particles of the seed meals (Fig. 3A). Thus, larvae could receive a more concentrated exposure of toxic isothiocyanate by feeding on seed meal particles. This was most apparent in the IG and PG seed meal treatments over 24-h exposure, where LC_50_ concentration is 75 and 72% less than the pure AITC and 4-HBITC treatments, respectively. The *Ls* and DFP treatments were more toxic than pure isothiocyanates with LC_50_ values that were 24 and 41% lower, respectively. Larvae from control treatment successfully pupated (Fig. 3B) while most larvae exposed to seed meal treatments failed to pupate and retarded larval development was apparent (Fig. 3C,D). In *Spodoptera* *littoralis*, isothiocyanates were associated with reduced growth and delayed development^79^.

**Table 5:** Computed amount of isothiocyanates (ppm) for the LC_50_ concentrations of seed meal when applied for control of *Ae.* *aegypti* larvae and evaluated at 24-h and 74-h continuous exposure.

Seed meal	LC_50_ (g/120 mL dH_2_O)	Glucosinolate (mg/120 mL dH_2_O)	Isothiocyanate (mg/120 mL dH_2_O)	Isothiocyanate (ppm)
24 h continuous exposure				
DFP	0.211	5.528	1.379	11.492
IG	0.115	4.538	1.692	14.104
PG	0.080	2.656	0.662	5.521
*Ls*	0.040	1.444	0.481	4.012
72 h continuous exposure				
DFP	0.111	2.908	0.725	6.045
IG	0.085	3.221	1.251	10.425
PG	0.051	1.693	0.422	3.519

*DFP* *Thlaspi* *arvense*, *IG* *Sinapis* *alba* (Ida Gold), *PG* *Brassica* *juncea* (Pacific Gold), *Ls* *Lepidium* *sativum*.

We did not examine the mechanisms responsible for the toxic effects of isothiocyanates on mosquito larvae. However, previous studies with red imported fire ants (*Solenopsis* *invicta*) revealed inhibition of glutathione S-transferases (GST) and esterases (EST) to be the main mechanisms of isothiocyanates bioactivity where AITC inhibited GST activity in red imported fire ants even at a low dose of 0.5 µg/mL^80^. Conversely, AITC inhibited acetylcholinesterase in maize weevil (*Sitophilus* *zeamais*) adults^81^. Similar studies should be conducted to identify the mechanisms of isothiocyanates activity in mosquito larvae.

We used a treatment of heat inactivated DFP to support the premise that the hydrolysis of plant glucosinolates to form active isothiocyanates as the mechanism for controlling mosquito larvae with mustard seed meals. The DFP-HT seed meal was not toxic at the tested application rates. Lafarga et al.^82^ reported that glucosinolates are sensitive to degradation by exposures to high temperature. The heat treatment was also expected to denature the myrosinase enzyme in the seed meal and prevent hydrolysis of the glucosinolates to form the active isothiocyanate. This is also validated by the results of the study of Okunade et al.^75^ indicating that myrosinase enzyme is temperature sensitive showing that myrosinase activity was completely inactivated when *B.* *juncea*, *B.* *nigra* and *S.* *alba* seeds were exposed to temperature above 80 °C. These mechanisms may have contributed to the loss of insecticidal activity of the heat treated DFP seed meal.

In conclusion, mustard seed meals and their three predominant isothiocyanates were toxic to mosquito larvae. Considering these differences between seed meal and chemical treatments, using seed meal may provide an effective method of delivery for mosquito control. Determining appropriate formulations and effective delivery system for improved potency and stability using seed meals are warranted. Our results demonstrate the potential use of mustard seed meals as an alternative to synthetic insecticides. This technology may provide an innovative tool for managing mosquito vectors. As mosquito larvae thrive in aquatic environments and seed meal glucosinolates are enzymatically catalyzed into active isothiocyanates when hydrated, application of mustard seed meals to mosquito infested water provides obvious control potential. While the larvicidal activity of the isothiocyanates varied (BITC > AITC > 4-HBITC), additional research is needed to determine whether combining seed meals with multiple glucosinolates would synergistically improve toxicity. This is the first study to document the insecticidal effect of defatted Brassicaceae seed meals and three bioactive isothiocyanates against mosquitoes. The results of this study open new opportunities by demonstrating that defatted brassica seed meals, the byproduct of seed oil extraction, can serve as promising larvicides for mosquito control. This information may facilitate further discovery of plant-based biocontrol agents to be developed as cheap, practical, and ecofriendly bioinsecticide.

## Data Availability

The datasets generated in this study and analysis of results are available from the corresponding author on reasonable request. All materials used in the study (insects and seed meals) were destroyed upon termination of the study.
